# Production and Quality Assessment of Fertilizer Pellets from Compost with Sewage Sludge Ash (SSA) Addition

**DOI:** 10.3390/ma18051145

**Published:** 2025-03-04

**Authors:** Paweł Cwalina, Sławomir Obidziński, Aneta Sienkiewicz, Małgorzata Kowczyk-Sadowy, Jolanta Piekut, Ewelina Bagińska, Jacek Mazur

**Affiliations:** 1 Department of Agri-Food Engineering and Environmental Management, Bialystok University of Technology, Wiejska St. 45E, 15-351 Białystok, Poland; p.cwalina@pb.edu.pl (P.C.); a.sienkiewicz@pb.edu.pl (A.S.); m.kowczyk@pb.edu.pl (M.K.-S.); j.piekut@pb.edu.pl (J.P.); 2 Institute of Forest Sciences, Bialystok University of Technology, Wiejska St. 45E, 15-351 Białystok, Poland; e.baginska@pb.edu.pl; 3 Department of Food Engineering and Machines, University of Life Sciences in Lublin, Głęboka St. 28, 20-612 Lublin, Poland; jacek.mazur@up.lublin.pl

**Keywords:** organic fertilizers, post-production residues, circular economy

## Abstract

This article examines the process of pressure agglomeration of garden waste compost mixed with sewage sludge ash (SSA) to produce granulated fertilizer material, using a flat die rotating compaction roller system. The study evaluated the effects of adding SSA at mass fractions of 0%, 10%, 20%, 30%, 40%, and 50% on the process of pelleting and the quality of pellets. Increasing the SSA content from 0% to 50% reduced the power demand of the pellet mill by 13.5% (from 4.92 kW to 4.25 kW), decreased the kinetic strength of the pellets by 0.7% (from 98.21% to 97.56%), and slightly increased the pellet density, by 2.6% (from 1641.17 kg·m^−3^ to 1684.09 kg·m^−3^). The high density of the pellets, i.e., over 1600 kg·m^−3^, indicates that they are of market quality. A chemical analysis revealed that SSA addition positively influenced fertilizer properties. A higher SSA content (up to 50%) decreased the nitrogen content (1.4% to 0.73%) but significantly increased the phosphorus content (0.32% to 2.67%). The potassium content remained stable, at approximately 1.3%. The process of co-pelleting also diluted the heavy metals present in SSA, reducing the final product’s lead and cadmium levels to meet the standards set for fertilizers. Although the SSA contained high levels of heavy metals (lead: 93.89 mg·kg_d.m._^−1^, cadmium: 11.28 mg·kg_d.m._^−1^), these elements were not detected in the compost. Co-pelleting of compost and SSA produces high-density, high-quality fertilizer pellets with favorable nutrient profiles and heavy metal contents, complying with regulatory standards. Moreover, by converting garden waste and SSA into valuable agricultural products, the process supports sustainable waste management. This study evaluated the impact of SSA additives on the composition and water absorption of the granulate, providing insights into its suitability as an eco-friendly fertilizer alternative and its potential implications for sustainable agricultural practices.

## 1. Introduction

Sewage sludge is a by-product of wastewater treatment [1]. Raw sewage sludge is characterized by a high moisture content of up to 98% [2], which means that this waste is produced in large quantities, both by volume and by weight. According to Wang et al. [3], the production of wet sludge in the USA, China, and Europe amounts to 240 metric tons (MT) per year. In contrast, according to the data reported by Ferrentino et al. [4], the production of sewage sludge globally, on a dry weight basis, is 45 million tons per year, with the production of such waste in the USA, China, and Europe varying between 18 and 33 million tons per year [5]. Due to population growth and stricter sludge regulations, sludge production is expected to increase.

Depending on the contents of pollutants present in sewage sludge, different methods are used to manage it. With the development of science and environmental awareness, some sewage sludge management methods have been restricted by regulations [6]. One such method, banned in 1998 by the Urban Waste Water Directive 98/15/EC [7], was the discharge of sewage sludge into the oceans. Another method is to use sewage sludge as a soil fertilizer; however, over time, restrictions were introduced for biodegradable waste, which limited the use of sewage sludge in this manner [8].

According to Brusselaers [9], incineration has become one of the more popular methods of sludge management, with 25% of the produced sludge being managed in this way. The process of sewage sludge incineration reduces the mass of the incinerated product by 70% and its volume by 70 to 90% [10,11]. Incineration results in a product called sewage sludge ash (SSA), which is considered the final by-product [10]. However, the sewage sludge disposal process does not end at this point, as SSA still contains a large amount of unburned material that can be managed. The current production of SSA is approximately 1.7 million tons, with the US, EU, and Japan being its main producers, and the production of ash from sludge incineration is expected to increase significantly [12,13].

The most common method of SSA management is its use in construction [14]. Numerous scientific studies have presented the possibility of managing SSA in the construction industry, in the form of a mortar additive or a cement substitute [15,16,17]. In addition, studies have found that ash produced through the incineration of sewage sludge can be used as a filler or a supplementary cementitious material (SCM) to replace cement. The use of SSA in construction can reduce the need for cement, the production of which causes high CO_2_ emissions [15]. As reported by Li et al. [18], the use of SSA in construction results in the immobilization of heavy metals present in the ash, thus reducing their emission into the environment.

Sewage sludge can also be used in agriculture, in countries where this is allowed by law. According to Brusselaers, up to 27% of the world’s sludge production is currently utilized in this way [9]. Although this is the main method used to manage sewage sludge in several western European countries, it is currently banned in countries such as Germany and the Netherlands [19]. As reported by Breda et al. [20], the use of sewage sludge as a fertilizer in tropical countries can have a positive effect on soil health, due to its lower fertility and organic carbon content [21,22]. However, it should be remembered that sewage sludge is a biological residue, characterized by a complex chemical composition depending on its origin, which can have a negative impact on the soil environment [23,24].

Another method of sewage sludge management and neutralization is composting or co-composting [25,26]. In recent years, a number of studies have been published focusing on the assessment of the environmental impact of compost-based granular fertilizers [27]. Studies [28,29,30] demonstrated the positive impact of the sewage sludge composting process on the content of pollutants and the amount of pathogens, among others. If the standards for the permissible levels of harmful substances in compost containing sewage sludge are met [31], this fertilizer can be considered environmentally safe enough to be used in agriculture. In addition, sewage sludge is a potential binder, and there is scientific evidence regarding its ability to improve the mechanical properties of pellets. On the other hand, it increases the ash content of the final product, as demonstrated in studies by Jiang et al. [32], who densified fir sawdust with sewage sludge. They found that as the ratio of fir sawdust to sewage sludge changed from 25% to 50%, the ash content increased from 4.28% to 13.01% [32].

The composting of garden waste is a widely used method to improve soil fertility and increase yields, especially in organic farming, where there is a concern about reducing the use of synthetic chemical fertilizers. The process not only contributes to soil quality but also promotes the long-term improvement of soil structure. Numerous studies have shown that compost provides essential nutrients such as nitrogen, phosphorus, and potassium, which are gradually released into the soil, ensuring that plants have long-term access to these elements [33,34]. In addition, compost improves soil water retention, which is particularly important in drought-prone areas, and increases the soil’s ability to retain nutrients, reducing the risk of leaching. Compost also stimulates microbial activity in the soil, promoting the growth of beneficial micro-organisms that are responsible for decomposing organic matter and improving soil structure, resulting in better plant growth, higher crop quality, and greater resistance to disease and pests [35].

Directive (EU) 2018/2001 of the European Parliament and of the Council of 11 December 2018 on the promotion of the use of energy from renewable sources sets mandatory levels of energy from renewable energy sources to be achieved, which includes energy generated from biomass such as municipal sewage sludge [36].

Although there are numerous papers on the management of SSA, few studies have focused on the combination of this waste and compost in the form of granular fertilizers. In particular, there is a lack of comprehensive analyses of their physicochemical properties, structural stability, and potential agronomic performance. The present work fills this gap by investigating the quality and stability of pellets formed from compost and SSA. The results obtained may have relevance for the development of sustainable fertilizer technologies, especially in countries aiming for efficient waste management and improved fertilizer practices in agriculture.

This study aimed to determine the most important material (raw material composition and moisture content of the compacted mixture) and process parameters during the pressure agglomeration of compost with the addition of ash produced through the incineration of sewage sludge. The research made it possible to create a fertilizer product in the form of pellets. The management of SSA in the presented manner opens up new possibilities for the neutralization of sewage sludge through pressure agglomeration. This paper presents a study of the effect of adding SSA at 0, 10, 20, 30, 40, and 50% to compost on the ash content; the contents of macro- and micronutrients, including heavy metals; and the water absorption capacity of the resulting granules.

## 2. Materials and Methods

### 2.1. Materials

**Figure 1:**
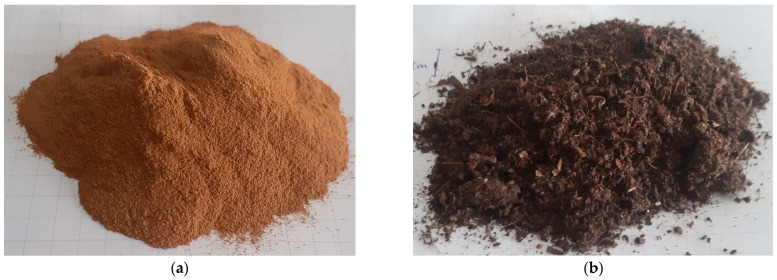
The raw materials used for the tests: (**a**) ash produced through the incineration of sewage sludge; (**b**) compost.

The raw materials used in the study to produce granulated fertilizer material included compost (Figure 1b) obtained from Przedsiębiorstwo Usługowo-Handlowo-Produkcyjne ‘LECH’ Sp. z o.o., based in Białystok, Podlaskie Voivodship, Poland.

The ash (SSA) (Figure 1a) was obtained from O-PAL Sp. z o.o., a company based in Skierniewice, Łódzkie Voivodship, Poland.

### 2.2. Determination of the Moisture Content of the Raw Materials

The moisture content of the analyzed materials was determined in accordance with PN-EN ISO 18134-3:2023-12 [37] using an AXIS ASG laboratory weighing machine (AXIS, Gdańsk, Poland). The measurement consisted in determining the moisture content using a weighing dryer by carrying out five repetitions at 105 °C. The final result was obtained by averaging the measurements.

### 2.3. Determination of the Granulometric Distribution of the Raw Materials

The granulometric distribution analysis of the raw materials was conducted following the PN-R-64798:2009 standard [38] using an LPz-2e laboratory sieve shaker (Multiserv Morek, Marcyporęba, Poland). This apparatus comprises a vibrating base and a series of sieves with square mesh sizes of 8 mm, 4 mm, 2 mm, 1 mm, 0.5 mm, 0.25 mm, 0.125 mm, and 0.063 mm.

### 2.4. Determination of the Bulk Density of the Raw Materials

The bulk density of the raw materials and pellets was tested in accordance with PN-EN ISO 17830:2016-07 [39] by filling a container of a known volume (with an effective inner diameter of 167 mm and an effective inner height of 228 mm). The bulk density was defined as the ratio of the mass of the material (weighed on an OHAUS AX224M (OHAUS Europe GmbH, Nänikon, Switzerland) analytical balance (with a measurement accuracy of ±0.1 mg)) to the volume of the container.

### 2.5. Determination of the Ash Content

The ash content of the raw materials was determined by burning the samples at 815 ± 15 °C, in accordance with PN-EN ISO 18122:2023-05 [40], and by using Equation (1):

(1) $$ash=\frac{m_{tp}-m_{t}}{m_{ts}-m_{t}}\cdot 100\%$$

where

*m_t_*—the weight of the crucible [g];

*m_ts_*—the weight of the sample before ashing [g];

*m_tp_*—the weight of the sample after ashing [g].

### 2.6. Determination of the Contents of Macronutrients

The contents of sodium, potassium, magnesium, and calcium were determined using atomic absorption spectrophotometry (ASA). This method made it possible to directly measure the contents of macronutrients in a solution of a previously mineralized sample. The tests were performed on a Thermo Scientific iCE3300 flame atomization-based atomic absorption spectrometer (Thermo Scientific, Waltham, MA, USA).

### 2.7. Determination of the Presence of Trace Elements and Heavy Metals

The tests for other elements such as aluminum, phosphorus, chromium, manganese, iron, cobalt, nickel, copper, zinc, arsenic, cadmium, lead, and mercury were performed using inductively coupled plasma mass spectrometry on an 8800 Triple Quadrupole ICP-MS spectrometer from Agilent Technologies (Agilent Technologies, Santa Clara, CA, USA).

### 2.8. Pressure Agglomeration Process

The production of pellets from a mixture of raw materials in the pressure agglomeration process—from the ash produced through the incineration of sewage sludge supplied by O-PAL Sp. z o.o. and the compost obtained from Przedsiębiorstwo Usługowo-Handlowo-Produkcyjne ‘LECH’ Sp. z o.o.—was carried out on an SS-5 test stand, presented in detail in the authors’ previous publication [41].

The process of producing granulated fertilizer material was carried out with the following constant quantities assumed during the tests:

- *w_m_* = 17—the moisture content of the mixture [%];
- *d_o_ =* 6—the mixture mass flow rate [mm];
- *Q_m_ =* 50—the mixture mass flow rate [kg·h^−1^];
- *n_r_ =* 270—the rotational speed of the densifying roller system [rpm];
- *h_r_* = 0.3—the gap between the rollers and the die [mm].

### 2.9. Determination of the Kinetic Strength of the Pellets

The kinetic strength of the pellets was evaluated according to the PN-EN ISO 17831-1:2016-02 standard [42] using a Holmen NHP100 tester (Tekpro, Mielec, Poland). The procedure was based on the methodology outlined in the authors’ previous study [43]. Before testing, the pellets were passed through a 5 mm mesh sieve to eliminate any impurities, such as broken pellets. A 100 g sample was then placed inside the test chamber, where the cascading motion within the air stream caused the pellets to collide with each other and with the perforated rigid surfaces inside the chamber. Once the test was completed, the sample was sieved again, and the remaining intact pellets were weighed. The kinetic strength (*P_dx_*) was determined using Equation (2):

(2) $$P_{dx}=\frac{m_{2}}{m_{1}}\cdot 100$$

where

*P_dx_*—the kinetic strength of the pellets [%];

*m*_1_—the mass of the sample before the test [kg];

*m*_2_—the mass of the sample after the test [kg].

### 2.10. Determination of the Physical and Bulk Density of Pellets

The bulk density of the pellets was measured using 10 representative samples. An EINHELL WSG-125E angle grinder (Einhell, Landau an der Isar, Germany) was used to smooth their edges. The prepared pellets were then measured with a caliper to an accuracy of 0.05 mm and weighed on an analytical balance with a precision of ± 0.001 g. The density (*ρ_g_*) was calculated using Equation (3):

(3) $$\rho_{g}=\frac{m_{g}}{V_{g}}$$

where

*ρ_g_*—the physical density of the pellets [kg∙m^−3^];

*m_g_*—the mass of pellets [kg];

*V_g_*—the volume of the tested pellets [m^3^].

The volume *V_g_* of the pellets was calculated using Equation (4):

(4) $$V_{g}=\pi \cdot r^{2}\cdot h$$

where

*V_g_*—the volume of the produced pellets [m^3^];

*r*—the radius of a pellet [m];

*h*—the height of a pellet [m].

The bulk density of the pellets was determined in accordance with PN-EN ISO 17830:2016-07 [39]. A container of known volume was filled with pellets and then weighed using an analytical balance (with a measurement accuracy of ± 0.1 mg). The bulk density was calculated as the ratio of the weight of the pellets to the volume of the container.

### 2.11. Determination of the Water Absorption Capacity of Pellets

The tests of pellet absorbability were carried out in accordance with PN-G-04652:1997 [44]. Pellet samples weighing 100 g were placed on a sieve and immersed in a vessel containing water. The water layer above the pellet sample was at least 30 mm thick. The pellet sample placed in the water was left for 24 h. After this time, the sieves were removed from the water and left for 2 min for the water to drain from the samples. After the pellets had dried, they were weighed on a balance to the nearest 0.01 g.

The water absorption capacity *WAC* of the pellets was calculated using Equation (5):

(5) $$WAC=\frac{{m_{2}-m}_{1}}{m_{1}}\cdot 100$$

where

*WAC*—the water absorption capacity [%];

*m*_1_—the mass of the pellet sample taken for the measurement [g];

*m*_2_—the mass of the pellet sample after water saturation [g].

### 2.12. Statistical Analysis

All results are expressed as mean values ± standard deviations (SDs) based on three replicates. A hierarchical cluster analysis (HCA) was used to construct a dendrogram that categorized the data into two clusters by analyzing the distances between object pairs. The dendrogram was generated using the Euclidean distance, with Ward’s method applied as the agglomerative criterion. A statistical analysis was conducted using Statistica 13.3 software (TIBCO Software Inc., Palo Alto, CA, USA).

The choice of hierarchical cluster analysis (HCA) for the statistical analysis was made due to its effectiveness in identifying groups with similar properties in data sets involving multiple variables. This method has been widely used in studies on the physicochemical characterization of fertilizer materials, as it allows the discovery of natural relationships between samples without the need to assume the number of groups in advance. This made it possible to classify the studied pellets more objectively based on their chemical and mechanical characteristics.

## 3. Results and Discussion

### 3.1. Moisture Content of Tested Raw Materials

The results for the moisture content of the tested raw materials and the prepared mixtures are shown in Table 1.

On the basis of the performed tests, SSA was found to have a minimum moisture content of 0.26%, while compost taken from the heap had a moisture content of 48.99%. As the moisture content of the fresh compost was too high, it was necessary to re-dry it, after which the content decreased to 7.51%.

According to Bajwa et al. [45], the ideal conditions for the process of pelleting to be performed at 25 °C are obtained for materials with a moisture content of 15–20% (in the wet state) or by preheating the matrix to 75 °C. Reaching the aforementioned temperature of the working system of the pellet mill is necessary to activate the binding components.

Based on the authors’ knowledge and experience, to obtain a product with suitable parameters, compost-based fertilizer mixtures pelleted on the SS-5 bench should have a moisture content of 17%. For this purpose, the mixtures with SSA were moistened before pelleting.

### 3.2. Bulk Density and Ash Content of Raw Materials

**Figure 2:**
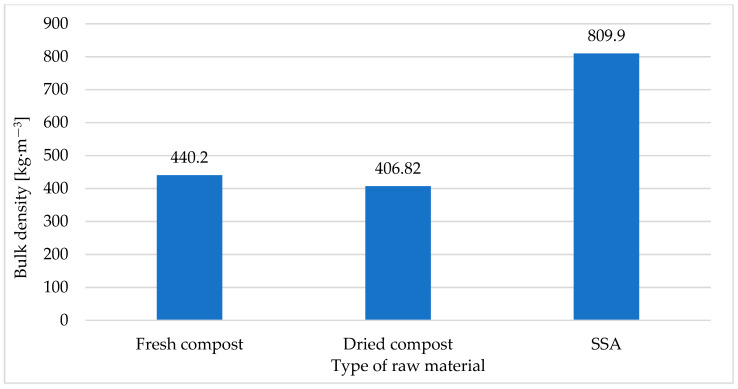
Bulk density of raw materials: SSA and compost.

Figure 2 shows the results of the tests of the bulk density of the raw materials, i.e., the ash produced through the incineration of sewage sludge (SSA), supplied by O-PAL Sp. z o.o., and compost obtained from Przedsiębiorstwo Usługowo-Handlowo-Produkcyjne ‘LECH’ Sp. z o.o.

Based on the conducted tests (Figure 2), it was found that the ash produced through the incineration of sewage sludge (SSA) supplied by O-PAL Sp. z o.o., used for the pressure agglomeration tests, was characterized by a bulk density of 809.90 kg·m^−3^, while the compost obtained from Przedsiębiorstwo Usługowo-Handlowo-Produkcyjne ‘LECH’ Sp. z o.o. (before the pressure agglomeration process, during the tests) was characterized by a much lower bulk density, i.e., 406.82 kg·m^−3^.

In its working state (i.e., right after being taken from the heap at ‘LECH’ Sp. z o.o.), the compost was characterized by a much higher bulk density of 1182.31 kg·m^−3^, compared to dried and shredded compost (440.20 kg·m^−3^).

### 3.3. Granulometric Distribution of Tested Raw Materials

Table 2 shows the granulometric distribution of the raw materials’ particles, i.e., the ash produced through the incineration of sewage sludge, supplied by O-PAL Sp. z o.o., and the compost obtained from Przedsiębiorstwo Usługowo-Handlowo-Produkcyjne ‘LECH’ Sp. z o.o., used in this study and subjected to sieve analysis.

The results show that the compost obtained from Przedsiębiorstwo Usługowo-Handlowo-Produkcyjne ‘LECH’ Sp. z o.o. contained small amounts of undesirable impurities (from the point of view of its use as a fertilizer) such as particles of foil, glass, and stones, as well as excessively large particles (e.g., pieces of branches or wood).

Table 2 shows that the fraction with a particle size of 1.00 mm (46.82%) and the fraction with a particle size of 0.50 mm accounted for the largest percentage share (approx. 35.29%) in the dried and shredded compost obtained from Przedsiębiorstwo Usługowo-Handlowo-Produkcyjne ‘LECH’ Sp. z o.o. The fractions with particle sizes of 0.25 mm, 0.125 mm, and 0.063 mm contributed slightly less. The fraction with a particle size of 2.00 mm accounted for the smallest percentage share (1.22%).

On the other hand, in the case of the ash produced through the incineration of sewage sludge supplied by O-PAL Sp. z o.o., the fraction with a particle size of 0.063 mm (61.02%) and the fraction with a particle size of 0.125 mm (approx. 30.43%) accounted for the largest percentage shares. The fraction with a particle size of <0.063 mm (4.66%) and the 0.25 mm fraction (3.89%) contributed slightly less.

### 3.4. Contents of Macronutrients

Table 3 shows the contents of selected macronutrients and sulfur in the tested raw materials and the produced pellets.

The nitrogen content of the tested raw materials remained at an unchanged level of 14.83 g·kg_d.m._^−1^ for SSA and 15.29 g·kg_d.m._^−1^ for compost. In a study conducted by Ajaweed et al. [46], the nitrogen content of the tested compost was 9.9 g·kg_d.m._^−1^. According to Mazur and Filipek-Mazur [47], depending on their composition, composts may contain 14.4 to 23.7 g·kg_d.m._^−1^ of nitrogen. According to Pulka et al. [48], the nitrogen content of sewage sludge is 43 g·kg_d.m._^−1^. Similar values were obtained by Krzywy et al. [49] (44 g·kg_d.m._^−1^), who studied municipal sewage sludge for its fertilizing properties.

The ash produced through the incineration of sewage sludge was characterized by a significantly higher phosphorus content compared to the tested compost. The phosphorus content of the tested SSA was 50.09 g·kg_d.m._^−1^, while the phosphorus content of raw sewage sludge was 12.6 g·kg_d.m._^−1^ [50]. However, this is almost twice as low as the value obtained by Latosińska [51], in whose study the tested sewage sludge ash contained 94.09 g·kg_d.m._^−1^. In contrast, in a study by Haustein et al. [52], the phosphorus content of dry SSA was 109.44 g·kg_d.m._^−1^. A similar value was obtained by Park et al. [53], who observed that the analyzed SSA was characterized by a phosphorus content of 108.79 g·kg_d.m._^−1^. Jama-Rodzeńska et al. [54] examined ash from sewage sludge incineration and reported that the SSA had a phosphorus content of 130 g·kg_d.m._^−1^. The phosphorus content of the tested compost was low, at 3.25 g·kg_d.m._^−1^. A study by Czop and Żydek [55] showed that the phosphorus content of green waste composts is 104.1 to 116.2 g·kg_d.m._^−1^. Cieszelczuk and Rosik-Dulewska [56] presented the differences between composts produced by professional composting plants and those made by individual farms, with the phosphorus contents being 2.75 and 4.53 g·kg_d.m._^−1^, respectively.

The potassium content of the tested ash (SSA) was 12.92 g·kg_d.m._^−1^. Similar potassium contents were obtained by Magdziarz et al. [57], in whose study, depending on the sample, the potassium content ranged from 9.71 to 23.57 g·kg_d.m._^−1^. A similar result (15.52 g·kg_d.m._^−1^) was also obtained by Latosińska [51]. On the other hand, raw sewage sludge is characterized by a potassium content of 0.84–28.47 g·kg_d.m._^−1^ [48,58]. In contrast, in a study by Godlewska and Becher [59], the potassium content of raw SSA was 4.06 g·kg_d.m._^−1^, which is low compared to the results obtained. The studied compost was characterized by the same potassium content as the ash (SSA), i.e., 13.05 g·kg_d.m._^−1^. In studies conducted by Cieszelczyk and Rosik-Dulewska [56], the potassium content of green waste compost ranged from 5.15 to 6.45 g·kg_d.m._^−1^.

The magnesium content of the tested ash produced through the incineration of sewage sludge was 7.77 g·kg_d.m._^−1^. Luyckx and Van Caneghem [60], who investigated the effect of the incineration temperature on the chemical composition of sludge, reported a magnesium content of SSA of 12.4–13.2 g·kg_d.m._^−1^, while Haustein et al. [52] obtained a magnesium content of SSA of 38.59 g·kg_d.m._^−1^. The tested compost contained a negligible amount of magnesium (1.55 g·kg_d.m._^−1^). In a study by Gondek and Kopec [61], the magnesium content of compost was 1.19 g·kg_d.m._^−1^.

The sulfur content of the tested ash (SSA) was 7.74 g·kg_d.m._^−1^, while the compost had a sulfur content of 1.57 g·kg_d.m._^−1^. In a study carried out by Bali et al. [62], the sulfur content of sewage sludge was 3.5 g·kg_d.m._^−1^.

### 3.5. Heavy Metal Contents

Table 4 shows the contents of heavy metals in the tested raw materials and the produced pellets.

In Poland, the contents of heavy metals in organic fertilizers are regulated by the Regulation of the Minister of Agriculture and Rural Development of 9 August 2024 on the implementation of certain provisions of the Act on fertilizers and fertilization (*Journal of Laws* 2024, item 1261) [63], which specifies the maximum contents of heavy metals in fertilizers.

The tested ash (SSA) was characterized by a chromium content of 207.16 mg·kg_d.m._^−1^, while the compost contained 22.79 mg·kg_d.m._^−1^ of chromium. The MRiRW regulation [63] allows a maximum chromium content of 100 mg·kg_d.m._^−1^. A study by Luyckx and Van Caneghem [60] showed that the chromium content of SSA increases with a rising temperature of sludge incineration. The chromium content of SSA was 139 mg·kg_d.m._^−1^ when incinerated at 550 °C and increased to 156 mg·kg_d.m._^−1^ when incinerated at 1100 °C.

The nickel content in both samples did not exceed the permissible content as specified in the Regulation of the Ministry of Agriculture and Rural Development [63]. The nickel content of the analyzed SSA was 59.56 mg·kg_d.m._^−1^. A study by Feng et al. [64] reported a nickel content of 63.6 mg·kg_d.m._^−1^. However, in a study by Latosińska [51], the nickel content of SSA was 29.01 mg·kg_d.m._^−1^. The nickel content of the analyzed compost was negligible (7.24 mg·kg_d.m._^−1^), while in a study by Mujtaba et al. [65], no nickel content was found in the tested green waste compost.

Copper and zinc are metals for which no standard contents have been established [61]. The contents of these elements in the SSA were 574.04 and 1944.83 mg·kg_d.m._^−1^, respectively. Similar results for copper were obtained by Luyckx and Van Caneghem [60], who reported a copper content of 256.66 mg·kg_d.m._^−1^ and a zinc content of 585.81 mg·kg_d.m._^−1^. In a study by Malarski et al. [66], the zinc content of the tested ash was 3159 mg·kg_d.m._^−1^, which is a value significantly higher than the results obtained in the described works. The contents of these elements in the analyzed compost were much lower, i.e., 37.50 mg·kg_d.m._^−1^ and 286.36 mg·kg_d.m._^−1^, respectively. The contents of these heavy metals in compost were also studied by Smolińska [67], who obtained much lower values for the analyzed composts, i.e., 1.35 mg·kg_d.m._^−1^ for copper and 0.85 mg·kg_d.m._^−1^ for zinc.

The arsenic content of the tested ash produced through the incineration of sewage sludge was 9.46 mg·kg_d.m._^−1^. In a study by Xiao et al. [68], the arsenic content of raw sewage sludge was 4.19 mg·kg_d.m._^−1^, while Kleemann et al. [69] reported an arsenic content of SSA of 5 mg·kg_d.m._^−1^. Similar values were obtained by Feng et al. [64], i.e., the arsenic content of the tested SSA was 6.64 mg·kg_d.m._^−1^ for ash produced through the incineration of sewage sludge at 500 °C and 4.37 mg·kg_d.m._^−1^ for ash produced through the incineration of sewage sludge at 1000 °C. No arsenic content was found in the tested compost.

The cadmium content in organic fertilizers can reach 5 mg·kg_d.m._^−1^ [63]. The tested ash (SSA) was characterized by a cadmium content of 11.28 mg·kg_d.m._^−1^ and therefore cannot be used as a fertilizer in its pure form. Studies indicate varying cadmium contents in ash (SSA), depending on the raw material from which it is produced. According to studies, the cadmium content of SSA can range from 0.5 to as much as 21 mg·kg_d.m._^−1^ [70,71,72]. In the case of the studied compost, no cadmium content was found. However, a study presented by Cieszelczuk and Rosik-Dulewska [56] showed a low cadmium content in the tested compost, ranging from 0.22 to 5.35 mg·kg_d.m._^−1^, depending on the method of production.

The final analyzed heavy metal was lead. Its contents were 93.89 mg·kg_d.m._^−1^ and 32.82 mg·kg_d.m._^−1^ in the SSA and compost, respectively. The MRiRW regulation [63] states that the permissible content of this element in organic fertilizers is 140 mg·kg_d.m._^−1^, which means that both the tested raw materials meet the standard. Raw sewage sludge is characterized by a lead content of 30.75 mg·kg_d.m._^−1^ [69]. In a study by Sun et al. [73], the lead content of SSA was much lower at 0.78 mg·kg_d.m._^−1^. In contrast, a study by Ottosen and Thornberg [74] reported a much higher lead content, ranging from 140 to 224 mg·kg_d.m._^−1^. In a study carried out by Voća’s team [75], who investigated the effect of MSS fertilization on soil, they found a lead content in the tested MSS of 66.5 mg·kg_d.m._^−1^. In addition, the findings of a study by Cieszelczuk and Rosik-Dulewska [56] showed that the lead content can vary depending on the raw material from which compost is produced and can range from 15.25 to 148.1 mg·kg_d.m._^−1^.

### 3.6. The Pelleting Process and the Characteristics of Pellets

**Figure 3:**
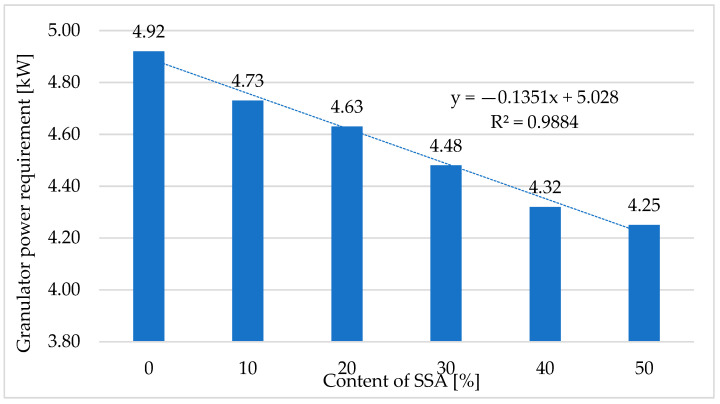
The relationship between the power demand of the pellet mill recorded during the pressure agglomeration of compost and the ash content of the pelleted mixture.

Figure 3 shows the results for the power demand of the pellet mill recorded during the pressure agglomeration of a mixture of ash produced through the incineration of sewage sludge and compost.

Based on the performed tests (Figure 3), it was found that the active power demand during compost pelleting was 4.92 kW at a mass flux rate of the mixture of 50 kg·h^−1^, resulting in a specific energy consumption of 98.4 kWh·t^−1^. Increasing the SSA content in the pelleted mixtures resulted in a reduction in the power demand of the pellet mill of 13.62% for the mixture containing 50% SSA. For the mixture containing 50% additive, the specific power consumption was 85 kWh·t^−1^.

The effect of the SSA additive on the active power demand *Ng* of the pellet mill during the pressure agglomeration of compost in the operating system of a flat die pellet mill is described by Equation (6):

(6) $$N_{g}=-0.1351\cdot Z_{SSA}+5.028$$

where

*N_g_*—the active power demand of the pellet mill [kW];

*Z_SSA_*—the SSA content [%].

Sarlaki et al. [76], who studied the environmental impact of pelleted compost, established a unit energy consumption that was approx. 15% lower, as the authors assumed an energy demand for compost pelleting of 83.15 kWh·t^−1^.

In contrast, in a study by Čajová Kantová et al. [77], the specific energy consumption during the process of pelleting of pine sawdust for energy purposes was 180.78 kWh·t^−1^, which indicates significantly lower costs of compost pelleting for fertilizer purposes compared to pressure agglomeration for energy purposes.

**Figure 4:**
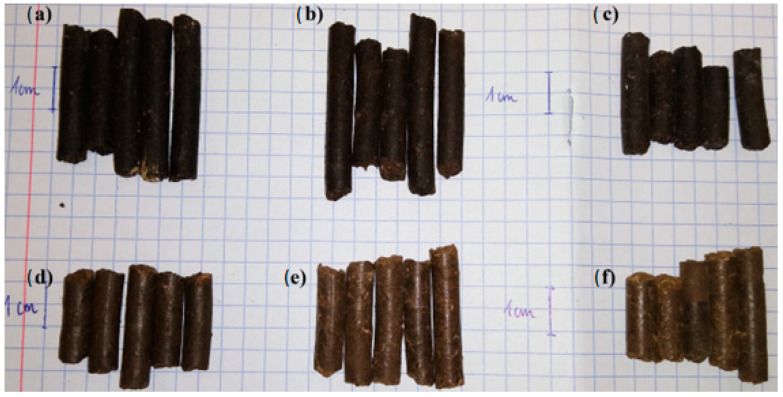
A view of pellets obtained in the process of pelleting (pressure agglomeration) of compost with the addition of ash produced through the incineration of sewage sludge in the following amounts: (**a**) 0%, (**b**) 10%, (**c**) 20%, (**d**) 30%, (**e**) 40%, and (**f**) 50%.

Figure 4 shows a view of the pellets obtained by the pelleting (pressure agglomeration) of compost with the addition of ash from sewage sludge incineration.

From the obtained test results (Figure 4), differences in the color of the produced pellets are evident. As the content of ash produced through the incineration of sewage sludge increased, the pellets became increasingly lighter, from completely black—for those obtained from compost alone (Figure 4a)—to increasingly grey.

### 3.7. Tests of the Pressure Agglomeration Process

Table 5 presents the results of tests of the power demand of the pellet mill, recorded during the pelleting (pressure agglomeration) of a mixture of ash produced through the incineration of sewage sludge, supplied by O-PAL Sp. z o.o., and compost obtained from Przedsiębiorstwo Usługowo-Handlowo-Produkcyjne ‘LECH’ Sp. z o.o., as well as the results of tests of the kinetic strength, physical density, and bulk density of the obtained pellets.

#### 3.7.1. Pellet Density

**Figure 5:**
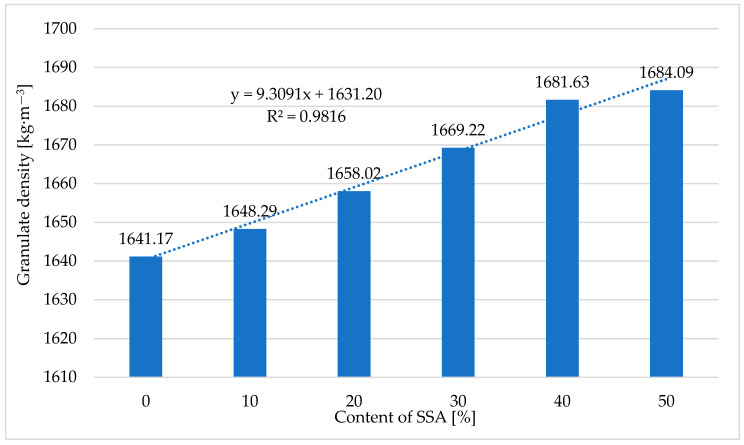
The relationship between the density of pellets obtained in the process of pressure agglomeration of compost and the ash content in the pelleted mixture.

Table 5 and Figure 5 present the results of tests of the density of pellets obtained in the pelleting process (pressure agglomeration) of a mixture of ash produced through the incineration of sewage sludge, supplied by O-PAL Sp. z o.o., and compost obtained from Przedsiębiorstwo Usługowo-Handlowo-Produkcyjne ‘LECH’ Sp. z o.o.

Based on the conducted tests (Table 5 and Figure 5), it was found that increasing the content of ash produced through the incineration of sewage sludge from 0 to 50% caused a slight increase in the density of the pellets, by approx. 2.6% (from 1,641.17 kg·m^−3^ to 1684.09 kg·m^−3^). Mieldazys et al. [78], who produced fertilizer pellets from composted animal excrement, obtained a density of pellets of 1497.32 kg·m^−3^. An equally high physical density of 1641.27 kg·m^−3^ was obtained by Papandrea et al. [79], who studied the effect of biochar addition on the physical properties of pellets produced from composts. The effect of the content of ash produced through the incineration of sewage sludge, *Z_SSA_*, in a mixture with compost obtained from Przedsiębiorstwo Usługowo-Handlowo-Produkcyjne ‘LECH’ Sp. z o.o. on the density of the pellets, *ρ_g_*, is described by Equation (7):

*ρ_g_* = 9.31 · *Z_SSA_* + 1631.2 (7)

where

*Z_SSA_*—the SSA content [%];

*ρ_g_*—the pellet density [kg·m^−3^].

#### 3.7.2. Bulk Density of Pellets

**Figure 6:**
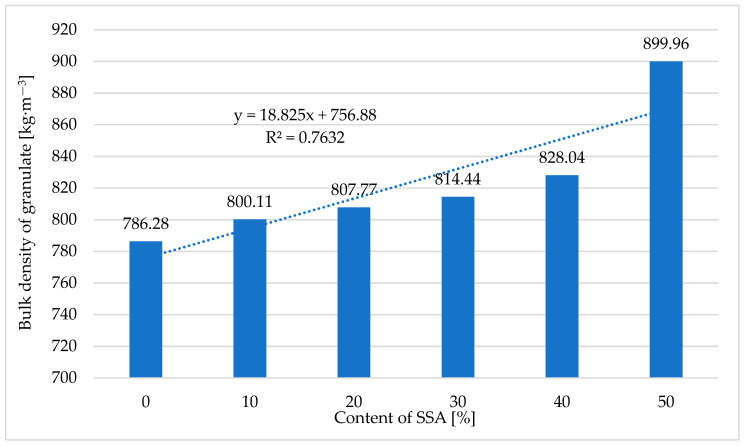
The relationship between the bulk density of pellets produced from compost in the process of pressure agglomeration and the ash content of the pelleted mixture.

Table 5 and Figure 6 present the results of tests of the bulk density of pellets obtained in the pelleting process (pressure agglomeration) of a mixture of ash produced through the incineration of sewage sludge, supplied by O-PAL Sp. z o.o., and compost obtained from Przedsiębiorstwo Usługowo-Handlowo-Produkcyjne ‘LECH’ Sp. z o.o.

Based on the conducted studies (Table 5 and Figure 6), it was found that increasing the content of ash produced through the incineration of sewage sludge from 0 to 50% caused the bulk density of the pellets to increase by approx. 14% (from 786.28 kg·m^−3^ to 899.96 kg·m^−3^). These values significantly exceed the value quoted in the standard [80], i.e., 600 kg·m^−3^. Pellets produced from compost are characterized by a high bulk density, which was confirmed in studies by Hettiarachchi et al. [81], who produced pellets from compost characterized by a bulk density of 730.7 kg·m^−3^. Pellets produced with the addition of biochar were characterized by a higher bulk density of 933.33 kg·m^−3^ [79]. The effect of the content of ash produced through the incineration of sewage sludge, *Z_SSA_,* in a mixture with compost obtained from Przedsiębiorstwo Usługowo-Handlowo-Produkcyjne ‘LECH’ Sp. z o.o. on the bulk density of the pellets, *ρ_g_*, is described by Equation (8):

*ρ_g_* = 18.83 *⸱ Z_SSA_* + 756.88 (8)

where

*Z_SSA_*—the SSA content [%];

*ρ_ug_*—the bulk density of pellets [kg·m^−3^].

#### 3.7.3. Kinetic Strength of Produced Pellets

**Figure 7:**
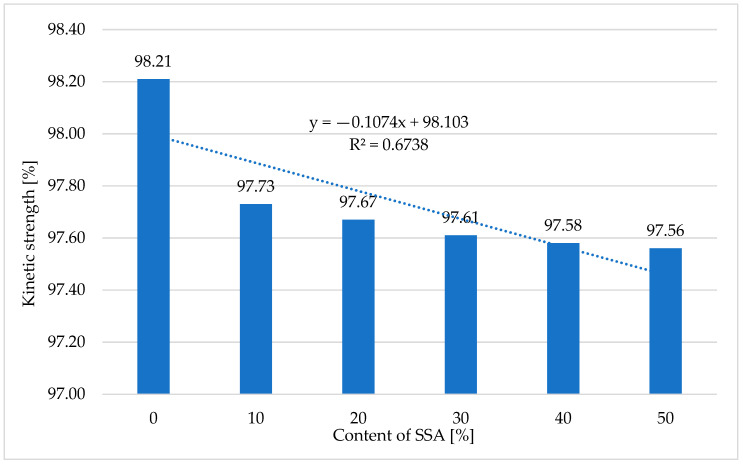
The relationship between the kinetic strength of pellets obtained in the process of pressure agglomeration from compost and the ash content of the pelleted mixture.

Table 5 and Figure 7 present the results of tests of the kinetic strength of pellets obtained in the pelleting process (pressure agglomeration) of a mixture of ash produced through the incineration of sewage sludge, supplied by O-PAL Sp. z o.o., and compost obtained from Przedsiębiorstwo Usługowo-Handlowo-Produkcyjne ‘LECH’ Sp. z o.o.

Based on the conducted studies (Table 5 and Figure 7), it was found that increasing the content of ash produced through the incineration of sewage sludge from 0 to 50% caused a slight decrease in the kinetic strength of the pellets, by approx. 0.7% (from 98.21% to 97.56%). Kinetic strength is one of the most important physical parameters of pellets, determining their resistance to unfavorable transport conditions [81,82]. According to the ISO 17225-2 standard [76], the minimum kinetic strength of pellets should be 97.5%.

The obtained high values of the kinetic strength of the pellets, *P_dx_*, assure that their quality is maintained even after a long period of storage. They also guarantee resistance to unfavorable (aggressive) transport conditions. The influence of the content of ash produced through the incineration of sewage sludge, Z_SSA_, in a mixture with compost obtained from Przedsiębiorstwo Usługowo-Handlowo-Produkcyjne ‘LECH’ Sp. z o.o., on the kinetic strength of the pellets, *P_dx_*, is described by Equation (9):

*P_dx_ =* −0.107 ⸱ *Z_SSA_* + 98.1 (9)

where

*Z_SSA_*—the SSA content [%];

*P_dx_*—the kinetic strength of the pellets [%].

### 3.8. Water Absorption Capacity of Produced Pellets

**Figure 8:**
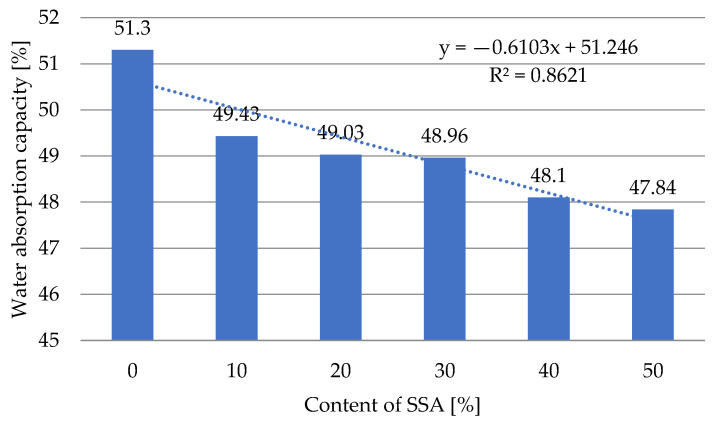
The relationship between the water absorption capacity of pellets produced from compost in the process of pressure agglomeration and the ash content of the pelleted mixture.

Based on the conducted tests (Figure 8), it was found that the highest water absorption (51.30%) was characteristic of the pellets produced from the compost obtained from Przedsiębiorstwo Usługowo-Handlowo-Produkcyjne ‘LECH’ Sp. z o.o. Ungureanu et al. [83] presented the results of tests of the absorption capacity of various pellets made from “lippie” grass, characterized by WAC levels of approx. 70%. The lowest WAC of approx. 28% was characteristic of pellets produced from tree rubber. Pitaktamrong and his team [84] investigated the effect of coating fertilizer pellets with alginate on their water absorption capacity. The addition of alginate in the coating from 1 to 3% resulted in an increase in WAC from 25–26% to 62–65%, which was important given the purpose of the fertilizer for fungal culture. Increasing the content of ash produced through the incineration of sewage sludge from 0 to 50% caused a decrease in the water absorption of the pellets by approx. 2.5%, i.e., from 51.30% to 47.84% (at a 50% ash content) (Figure 8).

The effect of the ash content, *Z_SSA_*, in a mixture with compost obtained from Przedsiębiorstwo Usługowo-Handlowo-Produkcyjne ‘LECH’ Sp. z o.o. on the water absorption capacity of the pellets, *WAC*, is described by Equation (10):

*WAC* = −0.6103 · *Z_SSA_* + 51.246 (10)

where

*Z_SSA_*—the SSA content [%];

*WAC*—the water absorption capacity [%].

### 3.9. Statistical Analysis of Obtained Results

**Figure 9:**
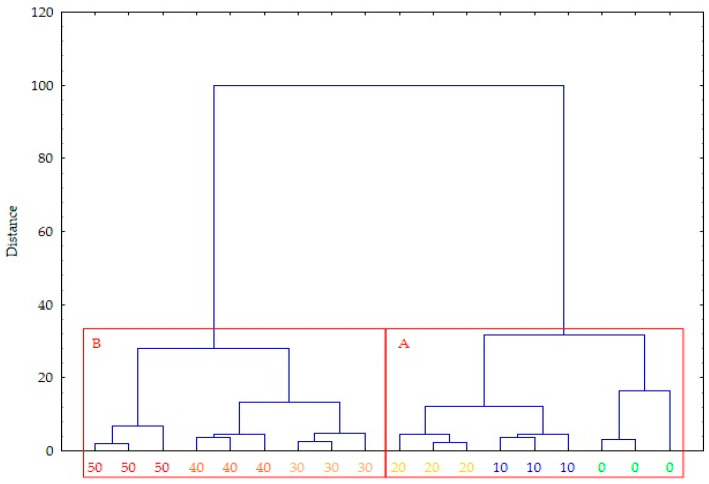
A dendrogram of the hierarchical cluster analysis of compost pellets with the addition of ash. Final partition: cluster A—ash contents of 0, 10, and 20%; cluster B: ash contents of 30, 40, and 50%.

The hierarchical cluster analysis effectively divided the tested pellets into two discrete clusters, denominated as A and B, based on the values of the material and process parameters of the pressure agglomeration of compost with the addition of ash produced through the incineration of sewage sludge (Figure 9). Cluster A, encompassing ash contents in pellets ranging from 0 to 20%, exhibits higher values of kinetic durability, water absorption, energy consumption, and N and K contents, relative to the group mean. This cluster also includes pellets with the highest values of these parameters at a 0% ash content. Conversely, cluster B, represented by ash contents in pellets from 30 to 50%, is characterized by distinctly higher values of density, bulk density, and Cr, Cu, Ni, Zn, Cd, P, As, Pb, Mg, and S contents. This cluster also includes pellets with the highest values of these parameters at a 50% ash content.

**Figure 10:**
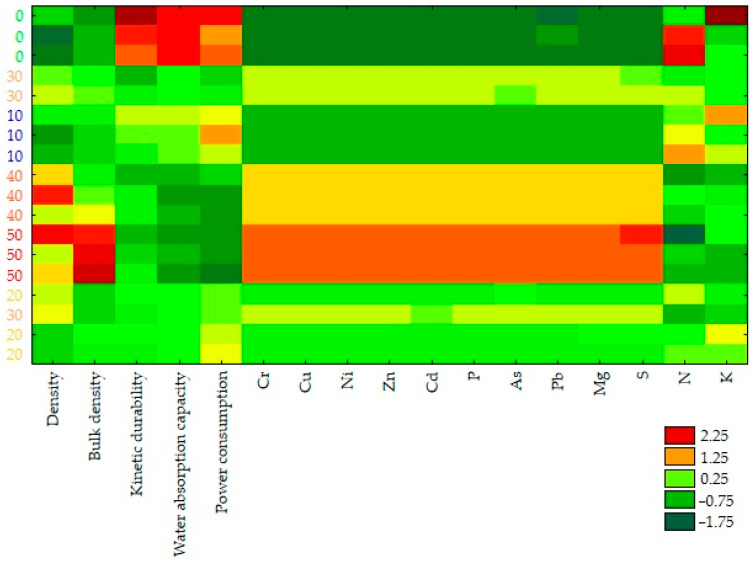
A graphical representation of the results of simultaneous grouping of objects (compost pellets with the addition of ash in the following amounts: 0%, 10%, 20%, 30%, 40%, and 50%) and features (material and process parameters of the pressure agglomeration).

In addition, when objects and features were simultaneously grouped, the analysis of the values of the material and process parameters of the pressure agglomeration of compost with the addition of ash produced through the incineration of sewage sludge revealed two distinctly dissimilar groups of objects (Figure 10). In the first group, pellets with an ash content ranging from 0 to 20%, characterized by higher values of kinetic durability, water absorption, energy consumption, and N and K contents (red) and lower values of density, bulk density, and Cr, Cu, Ni, Zn, Cd, P, As, Pb, Mg, and S contents (green), were grouped together. Conversely, the second group included pellets with an ash content ranging from 30 to 50%, characterized by higher values of density, bulk density, and Cr, Cu, Ni, Zn, Cd, P, As, Pb, Mg, and S contents (red) and lower values of kinetic durability, water absorption, energy consumption, and N and K contents (green).

## 4. Conclusions

Based on the conducted research, the following conclusions have been drawn:

1. Before the pressure agglomeration process, the compost must be dried so that the average moisture content of the compacted mixture of compost and ash does not exceed approx. 18–20%. Such a moisture content makes it possible to obtain a product (pellets) of satisfactory quality in terms of density and kinetic strength. A lower moisture content (below 12%) may cause the mixture to sinter on the die, which may ultimately lead to immobilization of the working system of the pellet mill. A higher moisture content (above 20%) will result in the deterioration of the physical parameters of the pellets.
2. The compost obtained from Przedsiębiorstwo Usługowo-Handlowo-Produkcyjne ‘LECH’ Sp. z o.o. contained small amounts of undesirable (from the point of view of its use as a potential fertilizer) inclusions, such as particles of foil, glass, or stones, as well as compost particles that were too large (from the point of view of the implementation of the pelleting process), e.g., pieces of branches or wood. Therefore, the compost should be crushed and then sieved before the pressure agglomeration (pelleting) process is carried out, to remove these undesirable inclusions.
3. The use of the addition of ash produced through the incineration of sewage sludge, supplied by O-PAL Sp. z o.o., allowed us to obtain granulated fertilizer material with good physical properties. Increasing the SSA content in a mixture with compost from 0 to 50% caused a slight increase in the density of the pellets, by approx. 2.6% (from 1641.17 kg·m^−3^ to 1684.09 kg·m^−3^). The addition of SSA also caused an increase in the bulk density of the pellets by approx. 14% (from 786.28 kg·m^−3^ to 899.96 kg·m^−3^).
4. Ash produced through the incineration of sewage sludge (SSA) was characterized by higher contents of phosphorus (50.09 g·kg_d.m._^−1^), magnesium (7.77 g·kg_d.m._^−1^), and sulfur (7.74 g·kg_d.m._^−1^) compared to the tested compost, which contained 3.25 g·kg_d.m._^−1^ of phosphorus, 1.55 g·kg_d.m._^−1^ of magnesium, and 1.57 g·kg_d.m._^−1^ of sulfur. The nitrogen and potassium contents of both the raw materials were similar, at 14.83 g·kg_d.m._^−1^ and 12.92 g·kg_d.m._^−1^ for SSA and 15.29 g·kg_d.m._^−1^ and 13.05 g·kg_d.m._^−1^ for compost. The pellets containing SSA exhibited increased contents of phosphorus, magnesium, and sulfur with an increase in the share of SSA in the mixture, which indicates the possibility of enrichment in these components.
5. Ash produced through the incineration of sewage sludge was characterized by higher contents of heavy metals (Cr, Ni, Cu, Zn, As, Cd, and Pb) compared to compost. The content of chromium in the SSA (207.16 mg·kg_d.m._^−1^) exceeded the permissible standard for organic fertilizers (100 mg·kg_d.m._^−1^). The co-pelletization of SSA with compost enabled a reduction in the Cr content, making it possible to use as much as 40% of the mixture without exceeding the set limit of 100 mg·kg_d.m._^−1^.
6. The low water absorption capacity, ranging from 47.84 to 51.3%, indicates that the produced pellets can be considered soil improvers with slow release into the soil. The use of ash produced through the incineration of sewage sludge, supplied by O-PAL Sp. z o.o., added to the compost, resulted in a decrease in the water absorption of the obtained pellets.

The obtained results indicate that the use of ash produced through the incineration of sewage sludge (SSA) in the co-pelleting process with compost enables the effective management of this waste, enriching the resulting granulated fertilizer material with valuable components such as phosphorus, magnesium, and sulfur. By using resources recovered from waste, this process helps to close the cycle of macronutrients in the environment. In the case of SSA, which cannot be used for agricultural purposes due to it exceeding the permissible contents of chromium (207.16 mg·kg_d.m._^−1^) and cadmium (11.28 mg·kg_d.m._^−1^), the process reduces its storage and makes it possible to create potential fertilizers with increased agricultural value. This aligns with the principles of a circular economy, promoting the sustainable management of raw materials and minimizing the negative impact on the environment.

The studies conducted herein focused mainly on the physicochemical properties of the obtained granules without assessing their long-term impact on the soil and crops. In the future, it is worth conducting field tests, which will allow for a more precise analysis of the fertilizers’ effectiveness in real conditions. Additionally, an important direction of further research may be the assessment of the stability and durability of granules in different storage conditions and their impact on soil microbiology.

## Figures and Tables

**Table 1:** Moisture content of tested mixtures.

Raw Material	Waste Content in Mixture [%]	Moisture ± SD [%]
SSA	10	17.01 ± 0.09 *
	20	16.92 ± 0.11 *
	30	16.88 ± 0.16 *
	40	16.99 ± 0.08 *
	50	16.73 ± 0.12 *
	100	0.26 ± 0.02
Compost in raw state (taken from heap)	100	48.99 ± 0.33
Dried compost	100	7.51 ± 0.11
	100	17.03 ± 0.15 *

*—moisture content of mixture before pressure agglomeration (after moistening).

**Table 2:** Granulometric distribution of tested raw materials.

Material	Share of Fraction [%]								
	8	4	2	1	0.5	0.25	0.125	0.063	<0.063
Compost	0.00	0.00	1.22	46.82	35.29	9.23	4.89	2.55	0.00
SSA	0.00	0.00	0.00	0.00	0.00	3.89	30.43	61.02	4.66

**Table 3:** Contents of selected macronutrients and sulfur.

Material	Nitrogen ± SD	Phosphorus ± SD	Potassium ± SD	Magnesium ± SD	Sulfur ± SD
	[g·kg_d.m._^−1^]				
SSA	14.83 ± 0.26	50.09 ± 0.41	12.92 ± 0.10	7.77 ± 0.06	7.74 ± 0.08
Compost	15.29 ± 0.24	3.25 ± 0.13	13.05 ± 0.11	1.55 ± 0.09	1.57 ± 0.04
Pellets containing SSA [%]					
10	15.24	7.94	13.03	2.18	2.19
20	15.19	12.62	13.02	2.80	2.80
30	15.15	17.30	13.00	3.42	3.42
40	15.11	21.99	12.99	4.04	4.04
50	15.06	26.67	12.98	4.66	4.65

**Table 4:** Heavy metal contents of tested raw materials and pellets.

Material	Cr ± SD	Ni ± SD	Cu ± SD	Zn ± SD	As ± SD	Cd ± SD	Pb ± SD
	[mg·kg_d.m._^−1^]						
SSA	207.16 ± 11.71	59.56 ± 0.48	574.04 ± 4.38	1944.83 ± 11.73	9.46 ± 0.29	11.28 ± 0.53	93.89 ± 0.82
Compost	22.79 ± 0.82	7.24 ± 0.43	37.50 ± 0.79	286.36 ± 2.01	0.00	0.00	32.82 ± 0.69
Pellets containing SSA [%]							
10	41.22	12.47	91.16	452.21	0.95	1.13	38.93
20	59.66	17.70	144.81	618.05	1.89	2.26	45.04
30	78.10	22.94	198.46	783.90	2.84	3.38	51.14
40	96.54	28.17	252.12	949.75	3.78	4.51	57.25
50	114.97	33.40	305.77	1115.60	4.73	5.64	63.36
Maximum concentration [63]	100	60	-	-	-	5	140

**Table 5:** Results of tests of kinetic strength, physical density, and bulk density of pellets.

SSA Content [%]	Density ± SD [kg·m^−3^]	Bulk Density ± SD [kg·m^−3^]	Kinetic Strength ± SD [%]
0	1641.17 ± 23.11	786.28 ± 11.18	98.21 ± 0.12
10	1648.29 ± 26.92	800.11 ± 14.28	97.73 ± 0.19
20	1658.02 ± 24.18	807.77 ± 10.89	97.67 ± 0.09
30	1669.22 ± 19.02	814.44 ± 17.67	97.61 ± 0.11
40	1681.63 ± 29.37	828.04 ± 18.43	97.58 ± 0.18
50	1684.09 ± 26.62	899.96 ± 23.82	97.56 ± 0.16

## Data Availability

Data are contained within the article.
